# Confidence Intervals for Population Allele Frequencies: The General Case of Sampling from a Finite Diploid Population of Any Size

**DOI:** 10.1371/journal.pone.0085925

**Published:** 2014-01-21

**Authors:** Tak Fung, Kevin Keenan

**Affiliations:** 1 National University of Singapore, Department of Biological Sciences, Singapore, Singapore; 2 Queen's University Belfast, School of Biological Sciences, Belfast, Northern Ireland, United Kingdom; 3 Queen's University Belfast, Institute for Global Food Security, School of Biological Sciences, Belfast, Northern Ireland, United Kingdom; University of Louisville, United States of America

## Abstract

The estimation of population allele frequencies using sample data forms a central component of studies in population genetics. These estimates can be used to test hypotheses on the evolutionary processes governing changes in genetic variation among populations. However, existing studies frequently do not account for sampling uncertainty in these estimates, thus compromising their utility. Incorporation of this uncertainty has been hindered by the lack of a method for constructing confidence intervals containing the population allele frequencies, for the general case of sampling from a finite diploid population of any size. In this study, we address this important knowledge gap by presenting a rigorous mathematical method to construct such confidence intervals. For a range of scenarios, the method is used to demonstrate that for a particular allele, in order to obtain accurate estimates within 0.05 of the population allele frequency with high probability (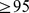
%), a sample size of 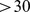 is often required. This analysis is augmented by an application of the method to empirical sample allele frequency data for two populations of the checkerspot butterfly (*Melitaea cinxia* L.), occupying meadows in Finland. For each population, the method is used to derive 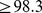
% confidence intervals for the population frequencies of three alleles. These intervals are then used to construct two joint 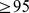
% confidence regions, one for the set of three frequencies for each population. These regions are then used to derive a 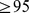
% confidence interval for Jost's *D*, a measure of genetic differentiation between the two populations. Overall, the results demonstrate the practical utility of the method with respect to informing sampling design and accounting for sampling uncertainty in studies of population genetics, important for scientific hypothesis-testing and also for risk-based natural resource management.

## Introduction

Spatiotemporal patterns of genetic variation among populations are often used to test hypotheses about processes underlying the patterns, such as selection, migration and genetic drift (e.g., [1], [2], [3], [4], [5]). This genetic variation captures differences in genetic structure among populations, where the genetic structure of a population is determined by the distribution of alleles among individuals in the population. The allele distribution of a population is the result of a range of biological and environmental processes acting on the population and also on surrounding populations within the same geographical region, which result in non-random mixing of gametes among individuals in all populations. Patterns in allele distributions among populations are generally assessed by consideration of variations in allele frequencies (e.g., [6], [7], [8], [9]).

Logistic and ethical constraints mean that in practice, a population is unlikely to be sampled in its entirety, such that the population allele frequencies have to be estimated using a subset of sampled individuals. For diploid organisms in a large population, it has been established that the frequency of an allele in a sample provides an unbiased estimate of the frequency in the population as a whole [10]. Thus, if samples of a given size are repeatedly taken from a large population, then the mean frequency of an allele in a sample converges to the population allele frequency as the number of samples increases. However, many studies only take a *single* sample from a population and present or use the resulting frequencies of alleles in the sample, without accounting for sampling uncertainty (e.g., [11], [12], [13], [14], [15], [16], [17], [18]). Therefore, these studies implicitly assume that the sample allele frequencies are close to the population allele frequencies, which is by no means guaranteed. Interpretation of findings from these studies is therefore complicated by the potential for large sampling uncertainty. Ideally, in this single sample case, uncertainty bounds for the population allele frequencies would be quantified, based on the sample allele frequencies.

A recent study by Hale et al. [19] used computer simulations to draw samples from four diploid populations, with allele frequencies based on four empirical datasets. Subsequently, they used the sample data to derive the means, variances and ranges of allele frequencies in samples of varying size, as well as the means, variances and ranges of indicators that are a function of allele frequencies (specifically, heterozygosity and F_ST_). Using these results, Hale et al. [19] concluded that a sample size of 30 is sufficient to accurately estimate population allele frequencies when using microsatellites. However, the authors did not quantify uncertainty bounds for the population allele frequencies using their sample data, such that their assessment of accuracy lacks a rigorous quantitative basis. Furthermore, for each sample size, sample frequencies were calculated using only 100 replicate samples, which may not give a good approximation of the true dispersion in the sample frequencies. This highlights a weakness of using a computational approach that lacks an underlying mathematical theory. Moreover, Hale et al. [19] did not consider the situation identified earlier, where one sample is taken and there is a need to quantify uncertainty of population allele frequencies using just the sample allele frequencies. For this situation, earlier studies have derived confidence intervals in order to capture uncertainty in the population allele frequencies. If a diploid population is of infinite size and is at Hardy-Weinberg equilibrium (HWE), then the frequency of an allele in a sample follows a binomial distribution [10]. Thus, Gillespie [20] proposed the use of the Wald confidence interval [21]. However, this interval only performs well for sufficiently large sample sizes – with small sample sizes, it is too short [22]. For the binomial distribution, other confidence intervals have been derived that do perform well for small sample sizes [22], [23], notably the Clopper-Pearson interval that was derived eight decades ago [24]. However, these only apply in the limited cases when the population is at HWE. Weir [10] proposed a confidence interval that allows for deviations from HWE, analogous to the Wald confidence interval. However, like the latter, it is expected to perform well only for sufficiently large sample sizes [10]. This is problematic because the accuracy at a particular sample size is unknown, so “sufficiently large” cannot be rigorously quantified. Moreover, all the confidence intervals considered only apply to cases when the population can be assumed to be of infinite size, i.e. when the population size is much larger than the sample size. This does not reflect the range of scenarios encountered in empirical research (e.g., [13], [25], [26], [27]).

In this study, we build on previous work by constructing confidence intervals for population allele frequencies for the general case where (i) the population is diploid, finite and can be of any size; (ii) the sample can take any size less than or equal to that of the population; and (iii) the population can deviate from HWE to any extent. These confidence intervals are guaranteed to contain the population allele frequency with a probability above a known threshold. The method derived for constructing these intervals is then used to calculate sample sizes required to achieve accurate estimates of population allele frequencies, under a range of scenarios. Here, accuracy is measured as the length of the confidence intervals. The sample sizes derived serve as a guide for determination of suitable sample sizes in future population genetic studies. In particular, we show that a sample size of 30 does not necessarily give accurate estimates, thus refining the conclusion of Hale et al. [19]. Lastly, we provide an example of how the method can be applied to microsatellite data for two populations of the checkerspot butterfly (*Melitaea cinxia* L.) [13], to derive confidence intervals for population allele frequencies and also for Jost's *D*, a measure of genetic differentiation between two populations that is a function of their population allele frequencies [9]. The data comes from a study [13] where it is unclear that the populations can be assumed to be of infinite sizes or at HWE. This example illustrates how the mathematical theory underlying our method can be used to quantify sampling uncertainty not only in population allele frequencies, but also in parameters that are functions of these frequencies, important for hypothesis-testing and also for natural resource management.

Overall, this study provides a rigorous mathematical quantification of sampling uncertainty in population allele frequencies, without the typically unrealistic constraints of assuming that a population has an infinite size and is at HWE. Thus, the results can be applied to a wide range of studies in population genetics.

## Methods

In order to construct confidence intervals for the general case of taking samples of any size from a diploid population of any size (larger than or equal to the sample size) and with any degree of deviation from HWE, the sampling distribution of the allele frequencies in this general case is first exactly specified. This distribution is then used to derive formulae specifying confidence intervals that contain the population allele frequencies with probability above a known threshold. Using these formulae, confidence intervals are derived for a range of archetypal scenarios, which are then used to calculate sample sizes that permit accurate estimates of population allele frequencies in these scenarios. In addition, the formulae are applied to a real scenario where samples are taken from two butterfly populations [13], to construct confidence intervals for the population allele frequencies of these two populations. The intervals are then used to derive a corresponding confidence interval for Jost's *D* [9].

### Derivation of sampling distribution of allele frequencies

Consider a population of *M* diploid individuals, from which a sample of *N* individuals is randomly drawn (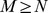). At the locus of interest, there are 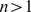 alleles, denoted by 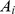, 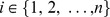. Let the population allele frequencies be denoted by 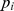, with the corresponding sample allele frequencies being denoted by 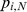. Also, let 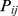 be the frequency of individuals in the population with alleles 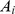 and 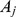 (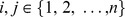), such that 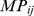 is the number of corresponding individuals in the base population. 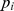 is related to 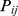 by the formula 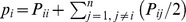. Here, 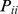 is a measure of the homozygosity of individuals in the population with respect to allele 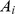. 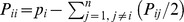, and thus 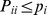. If 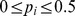, then the minimum value of 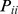 is 0, since all copies of allele 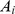 can be distributed among heterozygotes of allele 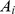. However, if 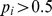, then this is not possible – there must be at least one homozygote of allele 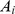. In this case, the minimum number of homozygotes is realized when all heterozygotes have a copy of allele 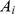, i.e. when 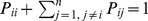. Rearranging this for 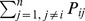 and substituting into 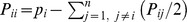 gives the minimum value of 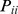 as 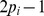. Thus, overall, 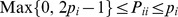.

The frequency of allele 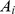 in the sample of size *N* is given by 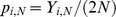, where 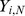 is the number of copies of allele 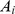 in the sample. Thus, the probability distribution of 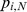 is the same as the probability distribution of 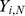 except with the *x*-axis scaled by a factor 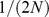. Denote the probability mass function (pmf) for 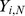 by 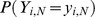. The range of 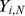 is 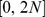, so 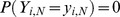 for 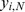 outside this range. Thus, for the following calculations, only 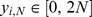 are considered. Now, 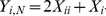, where 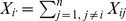 and 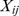 is the number of individuals in the sample with alleles 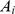 and 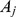. 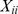, 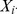 and 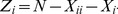 thus represent the number of individuals in the sample with two copies, one copy and no copies of allele 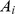, respectively. 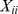, 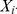 and 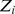 follow a multivariate hypergeometric distribution with pmf given by:
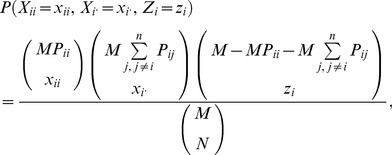
(1)where terms on the right-hand side in brackets are binomial coefficients. Since the number of individuals in the sample must equal *N*, 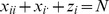. 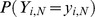 is the sum of 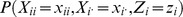 for all those biologically feasible combinations of 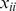, 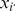 and 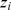 satisfying 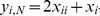. It will now be shown that this summation can be simplified to the sum of an expression that depends only on 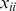 and 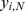, over all 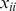 between lower and upper bounds that only depend on 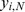. This considerably eases calculation of 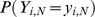. Firstly, the number of individuals in the sample with two copies, one copy or no copies of allele 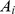 cannot exceed the corresponding numbers in the sampled population. This gives rise to three inequalities:
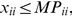
(2)

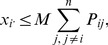
(3)

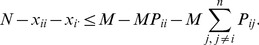
(4)Secondly, the number of individuals with two copies, one copy or no copies of allele 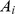 in the sample must be non-negative. This gives rise to three more inequalities:
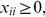
(5)

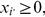
(6)

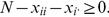
(7)Since 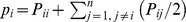 and 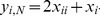, then 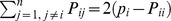 and 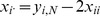. Thus, the inequalities (2)–(7) can be rearranged to obtain the double inequality:
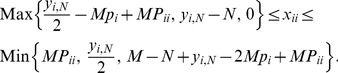
(8)It is noted that 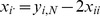, which means that 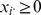 (inequality (6)) ensures that 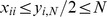, as required. Denote the upper and lower bounds in (8) by 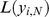 and 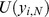 respectively. Then 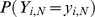 can be written as:
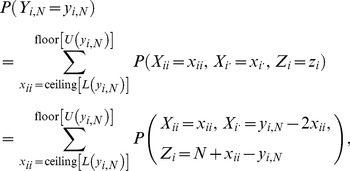
(9)where 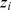 has been rewritten using 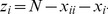 and 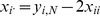, 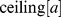 is the smallest integer larger than *a*, and 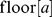 is the largest integer smaller than *a*. The ceiling and floor functions are introduced to ensure that 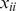 is an integer, which it must be to have a biological interpretation. 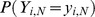 is equal to 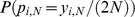, the pmf for 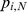, and thus defines the probability distribution of 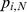. Equation (9) can be used to define the probability distribution of 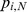 given only four parameters: *M*, 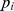, 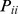 and *N*.

### Derivation of confidence intervals for sample allele frequencies

For given population size (*M*), population allele frequency (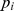), population frequency of homozygotes with allele 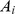 (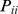) and sample size (*N*), the probability distribution for the sample allele frequency (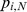) can be calculated exactly using equation (9). The mean value of this distribution is:
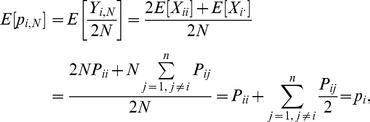
(10)as in the case of sampling from an infinite diploid population [10]. In equation (10), the expectation 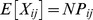 has been used, which follows from the fact that the 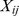
's, with 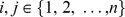, follow a multivariate hypergeometric distribution with parameters *M*, *N* and 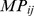 [28]. In addition, it can be proved that the variance of 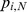 is
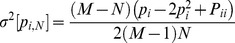
(11)(see File S1). The variance thus includes a standard finite correction factor 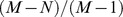 that tends to 1 as 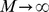, as required. Therefore, as 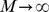, 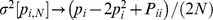, the variance in the case of an infinite population size [10]. The cumulative distribution function (cdf) for 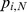 is specified by:
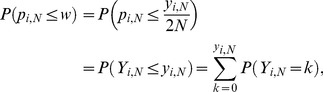
(12)where 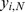 is an integer in the interval 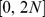. This cdf can be calculated using equation (9) and is a function of 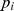. To construct a CI for 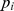 given *M*, *N* and 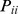, consider testing the null hypothesis 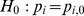 against the alternative 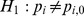 at significance level 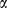 for an observed value of 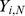, denoted by 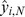. The null hypothesis is not rejected if using 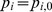, 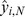 falls within an acceptance region defined by 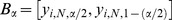, where 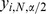 is the largest integer for which 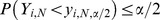 and 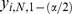 is the smallest integer for which 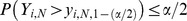. 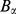 can be calculated using equation (12). One method of constructing a 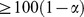
% CI for 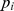 is to determine the set of values of 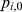 for which the null hypothesis is not rejected at significance level 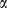, denoted by 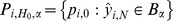, and defining the CI as
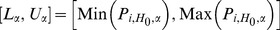
(13)
[29]. This hypothesis-testing approach corresponds to the “test-method” described by Talens [30], who applied it to construct CI's for parameters of the univariate hypergeometric distribution. If 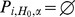, the empty set, then the acceptance region needs to be extended to include 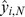 for at least one value of 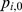. Thus, in this case, 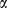 is decreased until 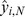 is in the acceptance region for at least one 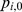 value.

The CI specified by equation (13) is not an exact 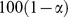
% CI because the distribution for 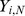 is discrete and because there may be some 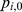 values between 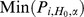 and 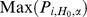 for which 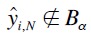, since 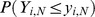 is not guaranteed to be a monotonic function of 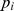 (see equations (9) and (12)). It is noted that for the probability parameter in a binomial distribution, Clopper and Pearson [24] derived 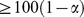
% CI's using an analogous method. Thus, for the case of a large population size relative to the sample size (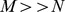) and HWE, where 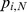 approximately follows a binomial distribution, CI's for 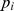 derived using our method would be virtually the same as Clopper-Pearson CI's. This can be verified by explicitly calculating and comparing CI's using the two methods – for example, if 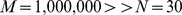 and 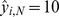, then both methods give the CI 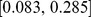 for 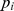.

In deriving equation (13), it was assumed that 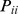 is known. However, 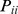 is generally unknown. In this case, a 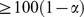
% confidence region (CR) can be derived for 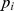 and 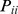 in an analogous way, by considering the null hypothesis 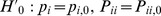. The CR can be derived by determining the set of vectors 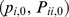 for which the null hypothesis is not rejected at significance level 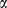. This set is denoted by 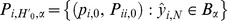, where 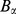 is as defined before. Using the CR, 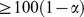
% CI's for 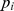 and 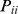 can be defined as
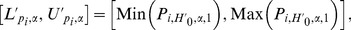
(14a)and
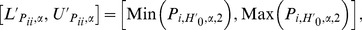
(14b)where 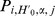 is the set of values consisting of the *j*th elements in the set of vectors 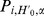. In determining 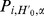, 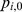 and 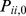 values over the biologically feasible ranges are tested. Since the number of copies of allele 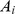 in the population must be at least the number found in the sample, 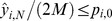. Also, the number of copies of all other alleles in the population must be at least the number found in the sample, 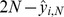; this constrains the maximum value of 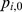 according to 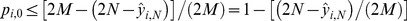. For a given value of 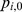, 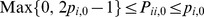, as determined earlier. If 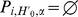, then the acceptance region is extended to include 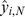 for at least one pair of values of 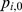 and 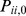, by decreasing 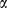.

CI's specified by equation (13) can be calculated given *M*, *N*, 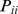 and 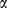, whereas those specified by equations (14a) and (14b) can be calculated given just *M*, *N* and 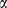. In this paper, computation of any CI's using these equations, incorporating the underlying formulae specified by equations (1), (8), (9) and (12), was carried out using the software package *Mathematica* v5.0 [31]. Supporting Webpage 1 provides *Mathematica* code for computation of the CI's and can be viewed at http://rpubs.com/kkeenan02/Fung-Keenan-Mathematica/. However, other software packages such as *MATLAB* [32] and *R* [33] could also be used to implement the formulae; indeed, an *R* version of the code is provided on Supporting Webpage 2 and can be viewed at http://rpubs.com/kkeenan02/Fung-Keenan-R/. All source code for the composition of Supporting Webpages 1 and 2, including the raw *Mathematica* and *R* code used, can be accessed at https://github.com/kkeenan02/Fung-Keenan2013/.

### Determining minimum sample sizes for accurate estimation of population allele frequencies

In studies of population genetics, it is desirable to obtain a sample allele frequency close to the population allele frequency with high probability [19], i.e. a short CI of high probability. Thus, under three representative scenarios, we calculate the minimum sample sizes required to obtain 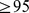
% CI's with lengths 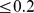 and 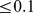 across all possible values of the observed sample allele frequency, 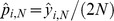. These minimum sample sizes are denoted by 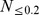 and 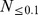 respectively. They are calculated by starting with 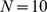, computing CI lengths for all possible values of 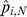 and then taking the maximum value. This is repeated for increasing *N* in increments of 10 until the maximum CI length becomes 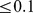. The *N* value with maximum CI length closest to 0.2 is then chosen, and then increased or decreased as necessary to find 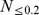. 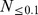 is derived analogously. Maximum CI lengths of 0.2 and 0.1 represent small maximum absolute errors in the estimated allele frequency of 0.1 and 0.05 respectively, if the estimate is taken as the mid-point of the CI. The first scenario examined, Scenario 1, is sampling from a population of 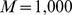 at HWE. 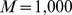 is larger than or on the same order of magnitude as the upper bound of the range of size estimates for 15/24 (63%) species populations collated by Frankham et al. [25], covering mammals, birds, insects and plants. Thus, 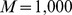 is taken to represent a large population. Later, *M* is varied over two orders of magnitude to consider populations that range from small to very large. Since there is a HWE, 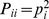. Also, in the sampled population, the number of homozygotes with allele *i*, 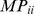, must be an integer. This restricts the number of values that 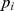 can take to 11 equally spaced values in the interval 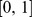 (starting from 0).

Scenarios 2 and 3 represent situations where HWE does not hold, such that 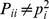. In Scenario 2, 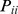 is assumed to take its minimum value, which is 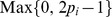, whereas in Scenario 3, 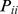 is assumed to take its maximum value, which is 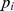. In comparison with Scenario 1, 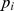 can now take a larger number of values – it can take the 2,001 equally spaced values in the interval 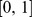 in Scenario 2 and the 1,001 equally spaced values in the interval 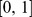 in Scenario 3. Since the variance of 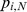 is an increasing function of 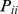 (equation (11)), CI's derived using 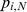 are expected to increase in length with 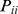 as well. This means that CI lengths and minimum sample sizes (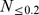 and 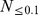) derived for Scenarios 2 and 3 are expected to encompass the entire ranges of possible values.

In the three scenarios examined, 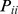 is specified as a function of 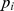, such that equation (13) can be used to calculate a CI for 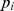 given a value of 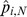. Calculation of this CI involves considering all possible values of 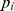 and determining under which values 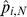 falls within the corresponding acceptance region (as described above). An alternative scenario, Scenario 4, is that the relationship between 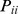 and 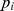 is unknown. In this case, equation (14a) has to be used to calculate a CI for 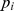 given a value of 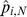, which involves considering all possible values of 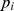 and 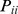. This results in a considerable increase in computation time; for example, when sampling 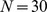 individuals from a population of 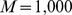, 2,001 values of 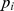 need to be considered but 1,002,001 combinations of 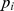 and 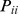 need to be considered, representing an increase in computational time by two orders of magnitude. However, for a given value of 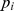, the maximum value of 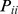 gives the highest variance for 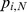 and is thus expected to maximize the length of the acceptance region within which 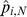 could fall within. Thus, CI's for 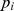 derived under Scenario 4 are expected to closely match those derived under the case of maximum homozygosity (Scenario 3). This can be verified by explicit calculation of the CI's – for example, given 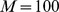, 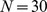 and 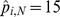, the CI's derived under Scenario 4 and Scenario 3 are 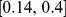 and 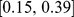, representing a difference in length of only 0.02. Similar results hold for 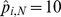 and 5. Therefore, results from Scenario 3 are used to approximate those for Scenario 4, obviating the need for long computational runtimes to explore all possible combinations of 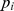 and 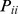.

Lastly, from equation (11), the variance of 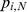 is an increasing function of *M*. Thus, CI lengths for 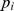 are expected to increase with *M*, which would result in increases in 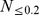 and 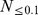. To test this explicitly, for a population at HWE and with 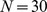, maximum lengths for the 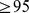
% CI for 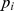 (across all values of 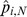) are calculated for 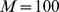, 250, 500, 750, 1,000, 2,500, 5,000, 7,500 and 10,000. This range corresponds to populations that are small to very large [25].

### Application to an empirical data set for checkerspot butterflies

To demonstrate how the theory developed can be used in practice, it is applied to microsatellite data for samples from two populations of the checkerspot butterfly (*Melitaea cinxia* L.) occupying meadows on the Åland Islands in Finland [13]. Specifically, for the *CINX1* locus, 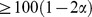
% CI's are calculated for the frequencies of alleles *A*, *B* and *C* for the Prästö and Finström populations, using the corresponding sample allele frequencies (Table 1 of Palo et al. [13]). For each population, a CI is not calculated for the population frequency of the fourth and final allele *D*, because this is fixed by the population frequencies of the first three alleles. To achieve consistency with the notation used in our study, henceforth, alleles *A*, *B*, *C* and *D* are referred to as alleles 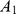, 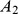, 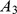 and 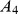 respectively. The sample size for the Prästö population is 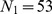, whereas that for the Finström population is 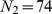 [13]. 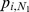 and 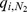, 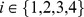, are used to denote the sample allele frequencies of 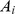 in the Prästö and Finström populations respectively. Similarly, 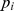 and 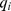 are used to denote the population allele frequencies of 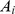 in the two populations, respectively. The two populations consist of two and seven subpopulations respectively. Thus, technically, the two populations can be referred to as metapopulations, although this terminology is not used in our study for clarity. The subpopulations form part of a total of about 536 subpopulations on the Åland Islands, with an estimated total size ranging from 35,000 to at least 200,000 [13]. Thus, the size of each subpopulation is assumed to be 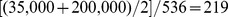, such that the Prästö and Finström populations are assumed to have a size of 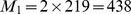 and 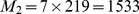 respectively. The observed sample allele frequencies in the two populations, denoted by 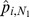 and 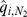 respectively, are taken directly from [13]. These are used to calculate the observed number of copies of allele 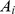 in each population, denoted by 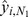 and 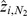 respectively, using the formulae 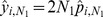 and 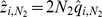. Due to rounding error in 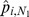 and 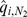 values from [13], 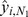 and 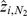 had to be rounded to the nearest integer. Since the true homozygosity of each allele in each population is unknown [13], conservative CI's are calculated assuming 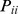 takes its maximum value of 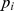 (this is expected to maximize the lengths of the CI's, as explained in the previous section). In addition, 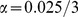 is chosen, such that a 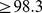
% CI is derived for each of the three population allele frequencies in each of the two populations. The reason for this choice of 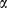 is because for each population, using the Bonferroni Inequality [34], the cubic region defined by the three CI's can be taken as a 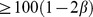
% confidence region (CR) for the three population allele frequencies, where 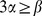. The choice of 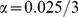 allows 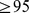
% CR's to be derived for each set of three population allele frequencies in the two populations.

To demonstrate how the theory developed in this paper can be used to derive CI's for genetic indicators that are a function of the population allele frequencies, the 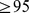
% CR's are used to calculate a 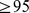
% CI for Jost's *D* for the *CINX1* locus and the Prästö and Finström butterfly populations. Jost's *D* is a measure of genetic distance between populations [9]. For the case of two populations, it is given by
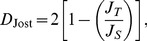
(15)where, using the notation in this example,
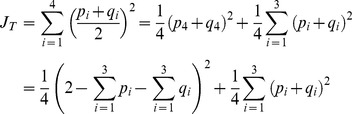
(16)and
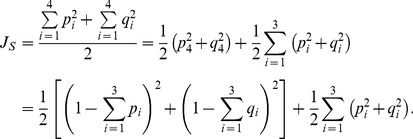
(17)A lower limit to a 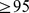
% CI for 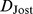 can be derived by minimizing the function in (15) given the constraints that the six population allele frequencies are contained within the two corresponding 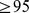
% CR's, and the two constraints 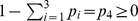 and 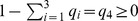. This lower limit is denoted by 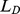. Similarly, the upper limit to a 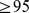
% CI for 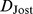 can be derived by maximizing the function in (15) under the same constraints. This is denoted by 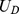. In this study, the “Minimize” and “Maximize” functions in *Mathematica* v5.0 [31] are used to compute 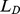 and 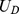, but corresponding functions may be used in other software packages, such as the “solnp” function in the “Rsolnp” *R* package. A 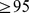
% CI for 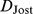 can then be defined as the interval 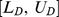. Supporting Webpage 1 provides *Mathematica* code that can be used to calculate 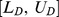 for the butterfly case study examined (http://rpubs.com/kkeenan02/Fung-Keenan-Mathematica/). Corresponding *R* code is presented on Supporting Webpage 2 (http://rpubs.com/kkeenan02/Fung-Keenan-R/).

## Results

### Maximum length of 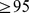
% confidence interval with increasing sample size

**Figure 1:**
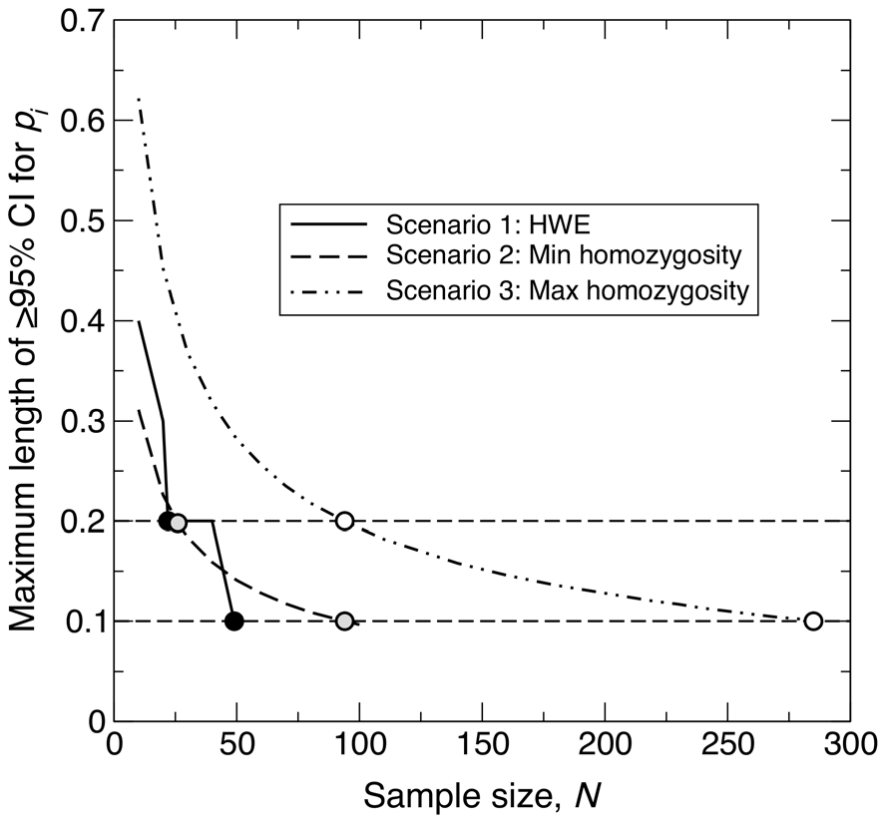
Change in maximum length of ≥95% confidence interval with increasing sample size. Graph showing how the maximum length of the 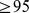
% confidence interval (CI) for the population frequency of an allele 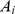 (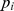) changes with increasing sample size *N*, when sampling from a diploid population of size 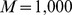. For a given *N*, the maximum CI length was derived by calculating CI lengths for all possible values of the observed sample allele frequency and then taking the maximum length. The three curves correspond to three scenarios where the population is (1) at Hardy-Weinberg equilibrium (HWE), (2) attains its lowest homozygosity value with respect to 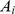, and (3) attains its highest homozygosity value with respect to 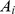. For each curve, the two filled circles represent the minimum *N* values required for the maximum CI length to reach values of 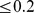 and 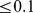. For visual guidance, the two dashed horizontal lines mark maximum CI lengths of 0.2 and 0.1. The exact values of *N* tested are described in *Methods*, and equation (13) was used to calculate the CI lengths.

For Scenario 1, where the sampled diploid population of size 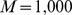 was at HWE, the maximum length of the 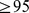
% CI for the population frequency of allele 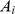, 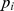, considering all possible observed values of the sample allele frequency, 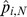, decreased non-linearly with sample size *N* (Figure 1). The minimum *N* required to achieve a maximum length 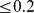, 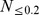, was 22, whereas the minimum *N* required to achieve a length 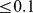, 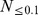, was 49 (Figure 1).

**Figure 2:**
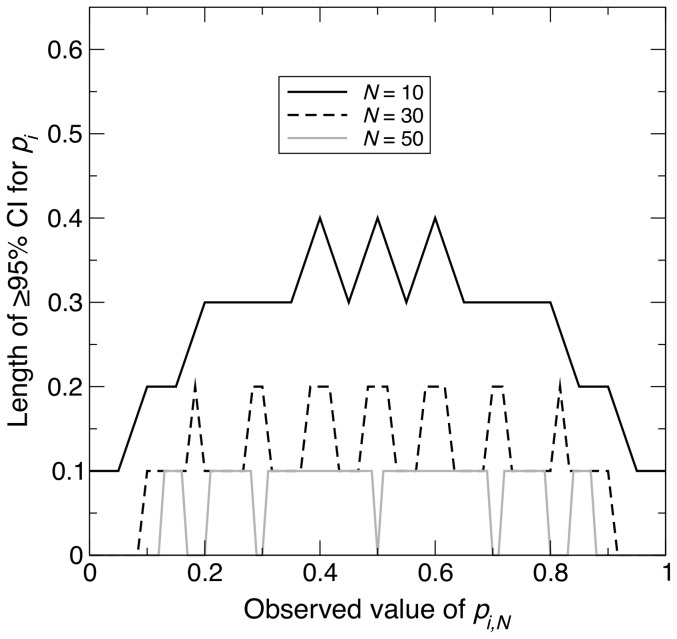
Change in length of 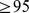
% confidence interval across different observed values, under Hardy-Weinberg equilibrium. For a sample of size *N* taken from a population of size 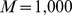 at Hardy-Weinberg equilibrium, graph showing the length of the 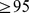
% confidence interval (CI) for the population frequency of an allele 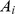 (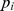) across all possible observed values of the sample allele frequency (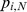). The three curves correspond to 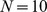, 30 and 50. Equation (13) was used to calculate the length of each CI.

For given *N* and 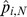, the CI for 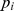 consists of all possible values of 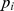 for which 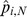 lies in the corresponding acceptance region, as described in *Methods*. This acceptance region is expected to increase with the variance of 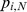, 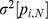. At HWE, the frequency of homozygotes of allele 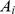, 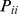, is equal to 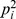; thus, according to equation (11), 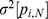 takes its highest values at intermediate values of 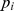. As a result, intermediate values of 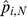 are likely to fall within the acceptance regions of more values of 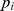, such that the corresponding CI lengths are generally longer. Simulation results for Scenario 1 were broadly in agreement with these theoretical expectations (Figure 2).

**Figure 3:**
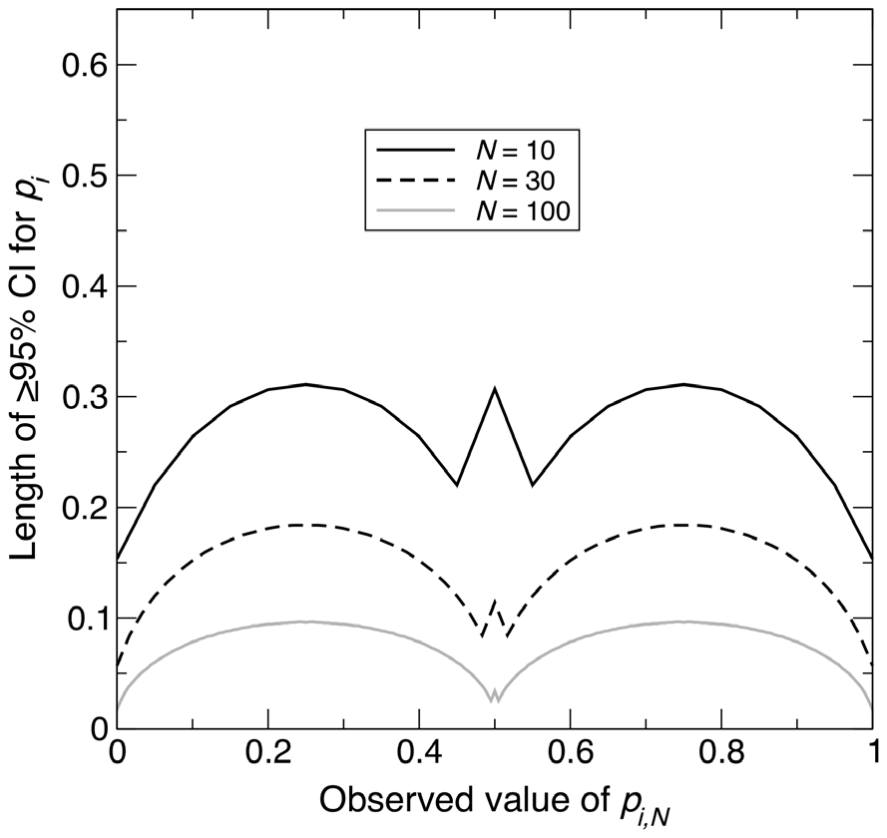
Change in length of 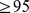
% confidence interval across different observed values, under minimum homozygosity. For a sample of size *N* taken from a population of size 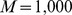 with the minimum homozygosity possible for an allele 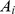, graph showing the length of the 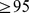
% confidence interval (CI) for the population frequency of 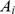 (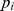) across all possible observed values of the sample allele frequency (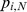). The three curves correspond to 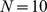, 30 and 100. Equation (13) was used to calculate the length of each CI.

In Scenario 2, where the population was no longer at HWE but attained its lowest 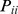, the maximum CI length also decreased non-linearly with increasing *N* (Figure 1). Compared with Scenario 1, 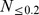 was slightly larger and 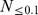 was larger by about a factor of two, taking values of 26 and 94 respectively (Figure 1). This is contrary to expectations that minimum homozygosity would give smaller 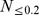 and 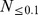 (see *Methods*). The reason is that although 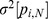 is smaller for given *N* and 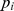 compared with the case of HWE, which would be expected to result in fewer 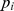 values for which a given 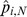 falls within the corresponding acceptance regions and hence a shorter CI, there are more possible values of 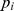 (2,001 compared with 11 – see *Methods*), which increased the number of 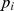 values for which 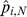 falls within the corresponding acceptance regions. For Scenario 2, 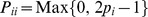, such that 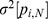 attains its highest values at 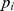 values close to 0.25 and 0.75 (equation (11)). Thus, for given *N*, values of 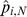 near 0.25 and 0.75 are expected to generally exhibit the longest CI lengths. Simulation results for Scenario 2 were broadly in agreement with these theoretical expectations (Figure 3).

**Figure 4:**
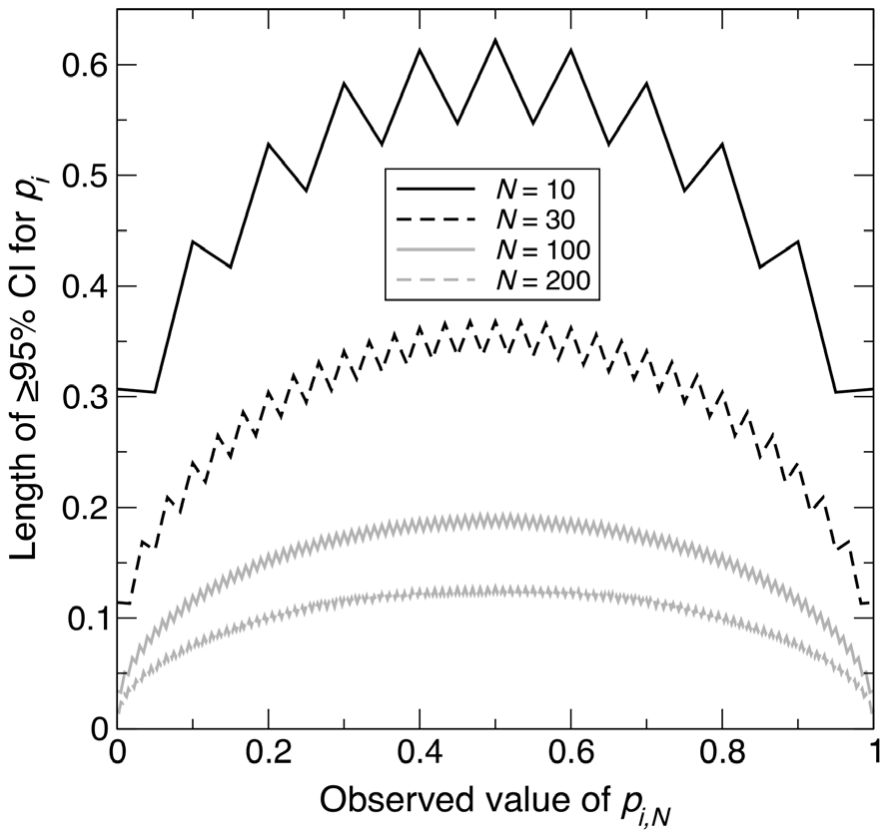
Change in length of 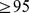
% confidence interval across different observed values, under maximum homozygosity. For a sample of size *N* taken from a population of size 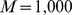 with the maximum homozygosity possible for an allele 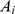, graph showing the length of the 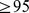
% confidence interval (CI) for the population frequency of 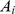 (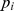) across all possible observed values of the sample allele frequency (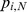). The four curves correspond to 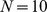, 30, 100 and 200. Equation (13) was used to calculate the length of each CI.

For the last scenario, Scenario 3, the population attained its highest 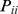, representing the opposite extreme to Scenario 2. As for the previous two scenarios, the maximum CI length decreased non-linearly with increasing *N* (Figure 1), but this time, 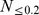 and 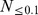 took values of 94 and 285 respectively. These values were approximately four and six times as large as the corresponding values in Scenario 1 (Figure 1). In Scenario 3, 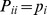, and equation (11) shows that intermediate values of 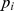 give the highest values of 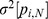. Therefore, as in Scenario 1, intermediate values of 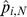 are expected to generally exhibit the longest CI lengths for given *N*. Again, simulation results were consistent with these expectations (Figure 4).

### Maximum length of 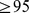
% confidence interval with increasing population size

**Figure 5:**
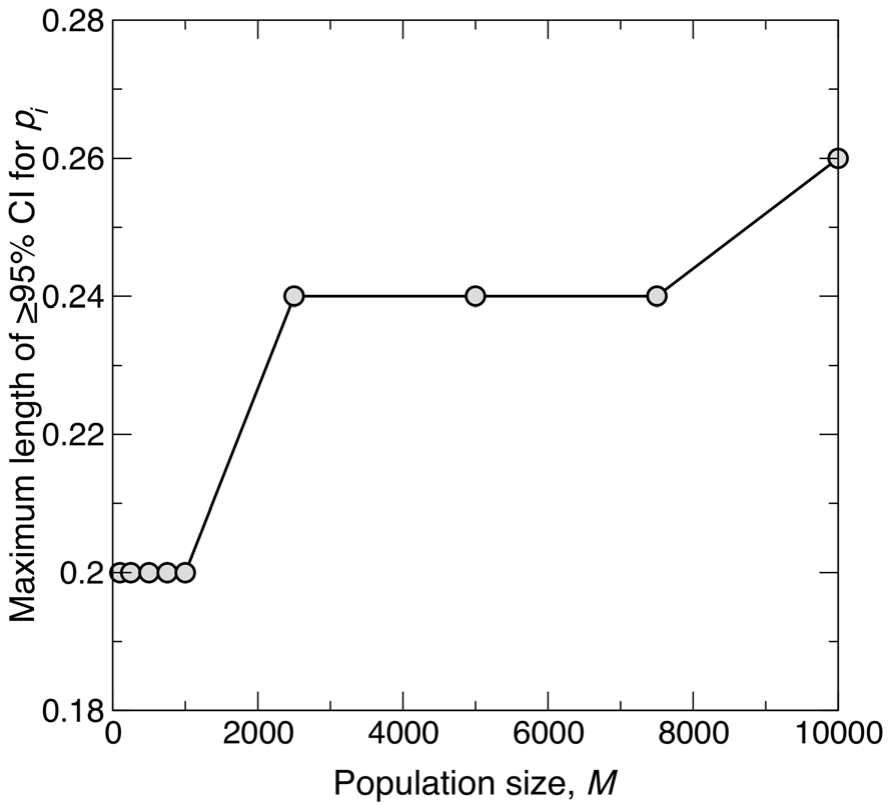
Change in maximum length of 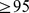
% confidence interval with increasing population size. Graph showing how the maximum length of the 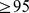
% confidence interval (CI) for the population frequency of an allele 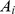 (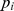) changes with increasing population size *M*, when taking samples of size 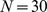. The population is at Hardy-Weinberg equilibrium. For a given *M*, the maximum CI length was derived by calculating CI lengths for all possible values of the observed sample allele frequency and then taking the maximum length. *M* values of 100, 250, 500, 750, 1,000, 2,500, 5,000, 7,500 and 10,000 were tested, as indicated by the filled circles.

When taking a sample of size 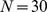 from a diploid population at HWE, the maximum length of the 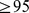
% CI for 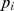, across all 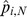, increased with population size *M* (Figure 5). As *M* was increased from a small value of 100 to 1,000, the maximum CI length remained the same at 0.2 (this is possible because there is nothing to prevent different values of *M* giving the same maximum CI length, using equation (13)). The maximum CI length increased when *M* was increased from 1,000 to a large value of 2,500, but only by 0.04. Thereafter, the length remained constant up to a very large value of 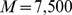, and only increased by a small amount of 0.02 with a further increase in *M* to 10,000. Thus, simulation results confirm the theoretical expectation that CI length increases with *M* (as explained in *Methods* – this expectation arises because 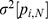 increases with *M*, according to equation (11)). However, the increase in the CI length was modest as *M* was increased over two orders of magnitude.

### 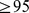
% confidence interval for Jost's D between two checkerspot butterfly populations

For the Prästö checkerspot butterfly population, the 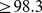
% CI's for the population frequencies for three of the four alleles at the *CINX1* locus were computed using equation (13) and data from Palo et al. [13], as described in *Methods*. The three alleles are denoted by 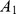, 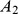 and 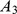 respectively, with the population frequencies denoted by 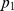, 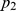 and 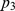 respectively. The three corresponding 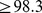
% CI's derived were 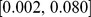, 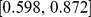 and 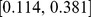 respectively. Using the same methodology, for the Finström population, the 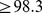
% CI's for the population frequencies of 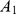, 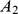 and 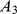 were calculated as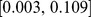, 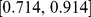 and 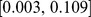 respectively.

The three 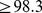
% CI's for the Prästö population were used to form a cubic region, which corresponds to a 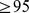
% CR for 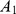, 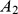 and 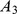 [34]. In the same way, the three 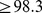
% CI's for the Finström population were used to form a 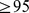
% CR for 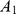, 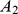 and 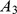. Within these two CR's, the maximum and minimum values of 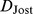 (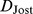 is given by equation (15)) were calculated and used to derive a 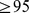
% CI for 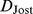, as described in *Methods*. This CI for 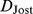 was derived as 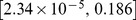. If 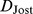 was calculated simply using the sample allele frequencies and equation (15), then only one value would have been obtained: 0.043.

## Discussion

In scientific studies that use sample data to estimate unknown population parameters, the sampling uncertainty in the estimates needs to be quantified in order to make reliable inferences on population processes captured by the parameters. This forms an essential part of scientific hypothesis-testing. Therefore, in studies of population genetics, it is essential to quantify the sampling uncertainty of key population parameters used to infer past and present evolutionary processes. These include allele frequencies, which are often used to quantify genetic variation among populations, thereby allowing hypotheses on processes driving this variation to be tested (e.g., [1], [2], [3], [4], [5]). However, many studies do not include sampling uncertainty for allele frequencies, instead presenting and/or using single point estimates based on one sample per population (e.g., [11], [12], [13], [14], [15], [16], [17], [18]). Thus, it is not possible to assess the accuracy of any inferences from these studies. This hinders not only the advance of scientific knowledge but also decision-making based on this knowledge, such as for sustainable management and conservation of natural resources. In this context, the work presented in this paper is valuable in that it provides a method of quantifying sampling uncertainty in allele frequencies for diploid populations, in the form of confidence intervals (CI's) containing true values with probability equal to or greater than a desired threshold.

The method presented pertains to the general case of a locus with *n* alleles, with a sample of size *N* taken from a population of size *M* and any degree of homozygosity with respect to the *n* alleles. In this case, the method allows construction of a CI for the population frequency of each allele, which can then be combined to create a joint confidence region (CR) for all the population allele frequencies at a given locus. It is noted that if more than one locus is considered simultaneously, then a joint CR for all population allele frequencies at all loci can be calculated by combining CI's in an analogous way. For the subcase of an infinite population size (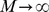) and a large sample size of 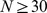, Weir [10] had proposed an approximate 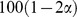
% CI for the population allele frequency of an allele 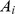, 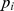. This is 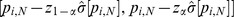, where 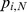 is the sample allele frequency; 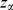 satisfies 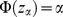, with 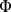 being the cumulative distribution function (cdf) of the standard Normal distribution; and 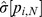 is an estimate of the standard deviation of 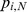 using sample data. 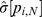 is specified by equation (11) with 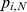 replacing 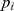 and 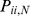, the sample frequency of homozygotes with allele 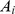, replacing 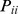, the corresponding population frequency of homozygotes. However, the accuracy of this CI depends both on how close 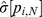 is to the true standard deviation 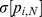 (specified by equation (11)) and how close the cdf of 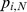 is to a Normal distribution with the same mean and variance. This accuracy has not been quantified [10] and thus, it is not known whether the CI actually contains 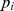 with a probability of at least 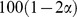, rendering its use problematic. In this study, we have rectified this problem by constructing a CI for 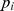 with probability coverage of at least 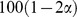, for the more general case where the population size can take any value larger than or equal to the sample size.

The method we derived was used to show that the sampling uncertainty in 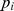, measured as the maximum length of the 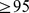
% CI for 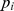 across all possible values of the observed sample allele frequency 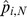, decreased non-linearly with *N* when sampling from a large population (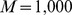) under three archetypal scenarios. These three scenarios represent the cases where the population (1) is at Hardy-Weinberg equilibrium (HWE), (2) has the lowest value of 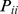 and (3) has the highest value of 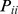. As expected from theory, for any given *N*, the maximum CI lengths for Scenario 3 were always greater than corresponding lengths in Scenarios 1 and 2. However, the maximum CI lengths for Scenario 2 was unexpectedly greater than that in Scenario 1 for some values of *N*, reflecting the greater possible number of values 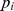 can take in a finite population with minimum homozygosity compared to one at HWE. This illustrates how the finite size of a population can give opposite trends to those obtained under an assumption of infinite size. According to theory and simulations, Scenario 3 gives CI lengths that closely approximate those in the case where 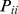 is unknown (see *Methods*). Thus, if 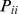 is unknown, CI lengths derived under Scenario 3 should be used. This was the approach used in the application of our method to sample data for two butterfly populations, discussed further below. On the other hand, if there is evidence that the population is at HWE, then the shorter CI's derived under Scenario 1 could be used.

Under the three scenarios examined, the non-linear decreases in sampling uncertainty with increasing *N* are consistent with results from the simulation study of Hale et al. [19], who found that the average difference between 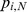 and 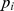 also exhibited non-linear decreases with *N*. However, Hale et al. [19] did not use their simulation results to quantify sampling uncertainty for the realistic situation where only one sample is taken from a population; this situation was considered in our study. Furthermore, as mentioned in the Introduction, for given *N*, they only used 100 samples to numerically construct the distribution for 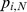, resulting in an incomplete distribution that may not closely reflect the true distribution. This highlights a weakness of a simulation-based approach without a rigorous mathematical underpinning, which is present in our approach. Hale et al. [19] concluded that *N* = 25–30 is sufficient to give accurate estimates of 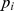, but this conclusion has to be interpreted in light of the limitations identified. Our results refine this conclusion by showing that across the three scenarios examined, *N* = 49–285 is required to ensure that, with a high probability of 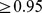, an estimate for 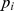 can be derived from any one sample that is within 0.05 of the true value; this corresponds to a CI of length 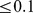. To ensure that the estimate is within 0.1 rather than 0.05, corresponding to a CI of length 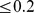, our results show that *N* = 22–94 is required. Thus, 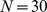 is not guaranteed to give “accurate” estimates of 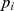 under all or most scenarios, and *N* values up to 10 times larger could be required. Decreasing the population size *M* from 1,000 would help to decrease sampling uncertainty, but results showed that decreasing *M* over two orders of magnitude from 10,000 to 100 only resulted in modest decreases in the maximum CI length of 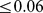, with no decrease when *M* was decreased from 1,000 to 100. Thus, the overall conclusion is that 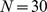 is often insufficient to guarantee accurate estimates of 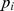, in the sense that 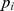 is within 0.05 or 0.1 of the estimate. Considering that alleles at highly polymorphic loci, such as microsatellites, often occur at population frequencies of 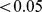 [35], it might be desirable to derive CI's for 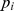 that are of length 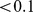. Thus, sample sizes even larger than the values found in our simulations might be required under some circumstances.

The application of our method to empirical data for two populations of the checkerspot butterfly [13] demonstrated how the underlying theory can be applied to construct joint 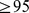
% CR's for the population frequencies of multiple alleles at a single locus. These CR's were then used to construct a 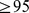
% CI for Jost's *D*, which measures genetic differentiation between the two populations. This illustrates how our method can be used to quantify sampling uncertainty in genetic indicators that are a function of population allele frequencies, thus facilitating hypothesis-testing and also risk-based natural resource management. In the example considered, the single point estimate of Jost's *D* using the sample allele frequencies was about four times lower than the upper bound of its 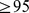
% CI. Thus, use of the single point estimate without accounting for sampling uncertainty could lead to misleading conclusions. The effectiveness of any management measures based on such conclusions would be compromised, hindering the achievement of objectives related to conservation or sustainable use. Our example therefore highlights the practical utility of the method that we have derived.

In conclusion, we have presented a rigorous mathematical method for quantifying sampling uncertainty in estimates of population allele frequencies, for a general case that has hitherto not been analyzed. In addition, we have demonstrated its practical application in informing sampling design and determining uncertainty in genetic indicators. Thus, the method derived advances both theory and practice, with broad implications for a range of disciplines, including: conservation genetics, evolutionary genetics, genetic epidemiology, genome wide association studies (GWAS), forensics and medical genetics. In particular, the method provides exact answers to the question of how many individuals need to be sampled from a population in order to achieve a given level of accuracy in estimates of population allele frequencies. This is a question that has rarely been studied before [19], despite its important practical implications. Previous studies have derived sample sizes required to sample, with high probability, at least one copy of all alleles at a locus above a given frequency [35], [36], [37], but these do not correspond to sample sizes required to achieve accurate estimates of population allele frequencies. Derivation of the latter requires explicit quantification of sampling uncertainty, as we have done in this study.

### Possible future extensions

The CI's and CR's constructed using our method are conservative in the sense that they contain the true values with a probability equal to *or* greater than a desired threshold. This conservative property is useful in hypothesis-testing if there is a need to decrease the probability of obtaining a false positive below a certain threshold. However, researchers would ideally like to construct CI's and CR's containing true values with a known probability, not just with probability at or above a known threshold. Therefore, future research could attempt to tighten the intervals and regions that we have derived, ideally until they cover a known probability. For example, Cai and Krishnamoorthy [23] devised a method (using their “Combined Test” approach) that was shown to give shorter CI's for the probability parameters of both the binomial and univariate hypergeometric distributions, when compared with a hypothesis-testing approach analogous to the one used in this paper. If their method could be extended to the population allele frequency parameter for the more complex distribution used in this paper (equation (9)), based on a multivariate hypergeometric distribution (equation (1)), then this might result in tighter CI's for population allele frequencies when sampling from a finite diploid population.

In addition, the CI's derived in our paper were designed to quantify the uncertainty in population allele frequencies that arises from taking a random sample of a finite diploid population, which can exhibit any degree of relatedness. The “random” refers to equal probability of choosing individuals that may be of any type, which does not imply that once an individual of a particular type has been sampled, the next individual sampled is equally likely to belong to any of the types (i.e., does not imply individuals in the population or sample are unrelated). Relatedness among individuals in the population is implicitly included within the population frequencies of the different genotypes, and is thus accounted for when calculating the sampling distribution for the population frequency of an allele 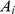, as specified by equation (1). However, this representation does not give an explicit quantification of the degree of relatedness among individuals in the population, for example using kinship coefficients. DeGiorgio and Rosenberg [38] used such coefficients to derive an unbiased estimator of heterozygosity (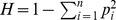, for a locus with *n* alleles) in the case of sampling from a population of diploid individuals that could be related, and DeGiorgio et al. [39] extended these results to the case of individuals with arbitrary ploidy. In their calculations, the number of copies of allele 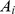 in each sampled individual *k* was treated as a random variable and the covariances of these variables were then related to the kinship coefficients. This is different to calculations in our paper, where the number of sampled individuals with alleles 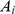 and 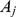 was treated as a random variable (see *Methods*). The method in this paper might be revised by considering the number of copies of allele 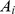 for each sampled individual instead, following [38], [39]. This could allow explicit quantification of relatedness in the context of deriving CI's for the population allele frequencies.

## Supporting Information

File S1 **Derivation of the variance of the sample allele frequency.**
(PDF)
