# Supplementary material for: Confidence Intervals for Population Allele Frequencies: The General Case of Sampling from a Finite Diploid Population of Any Size
Source: PLoS One. 2014 Jan 21;9(1):e85925. doi: 10.1371/journal.pone.0085925 (PMC3897575; doi:10.1371/journal.pone.0085925)
Supplement: File S1 — Derivation of the variance of the sample allele frequency. (PDF) [file pone.0085925.s001.pdf]

Supporting Information S1

Derivation of the variance of the sample allele frequency

Consider a population of *M* diploid individuals, from which *N* individuals are randomly sampled. Let the locus of interest have $n > 1$ alleles, denoted by A_i, $i \in \{1, 2, \ldots, n\}$. Also, let P_{ij} be the frequency of individuals in the population with alleles A_i and A_j, with $i, j \in \{1, 2, \ldots, n\}$. Thus, MP_{ij} is the number of individuals in the population with alleles A_i and A_j. The frequency of allele A_i in the population is denoted by p_i, whereas that in the sample is denoted by $p_{i,N}$ (a random variable). $p_{i,N} = Y_{i,N} / (2N)$, where $Y_{i,N}$ is the number of copies of allele *i* in the sample of size *N*. $Y_{i,N} = 2X_{ii} + \sum_{j=1, j \neq i}^{n} X_{ij}$, where X_{ij} is the number of individuals in the sample with alleles A_i and A_j. $Y_{i,N}$ can be written as $Y_{i,N} = \sum_j a_j X_{ij}$, where $a_j = 2$ for $i = j$ and $a_j = 1$ for $i \neq j$. Using the formula for the variance of a sum of dependent variables, the variance of $Y_{i,N}$ is equal to:

$$\sigma^2\left[Y_{i,N}\right] = \sum_j a_j^2 \sigma^2\left[X_{ij}\right] + \sum_{j \neq k} \sum_k a_j a_k \mathrm{Cov}\left[X_{ij}, X_{ik}\right], \qquad \text{(S1)}$$

where σ^2 and Cov denote the variance and covariance respectively. The X_{ij}'s, with $i, j \in \{1, 2, \ldots, n\}$, follow a multivariate hypergeometric distribution with parameters *M*, *N* and MP_{ij}. Thus,

$$\sigma^2\left[X_{ij}\right] = \left(\frac{M-N}{M-1}\right) NP_{ij}\left(1-P_{ij}\right), \qquad \text{(S2)}$$

and

$$\mathrm{Cov}\left[X_{ij}, X_{ik}\right] = -\left(\frac{M-N}{M-1}\right) NP_{ij}P_{ik} \qquad \text{(S3)}$$

[1]. Thus,

$$\sigma^2\left[Y_{i,N}\right] = \left(\frac{M-N}{M-1}\right) N \left(\sum_j a_j^2 P_{ij}\left(1-P_{ij}\right) - \sum_k \sum_{j \neq k} a_j a_k P_{ij} P_{ik} \right)$$

$$= \left(\frac{M-N}{M-1}\right) N \left(a_i^2 P_{ii}\left(1-P_{ii}\right) + \sum_{j \neq i} a_j^2 P_{ij}\left(1-P_{ij}\right) - 2\sum_{j \neq i} a_i a_j P_{ii} P_{ij} - \sum_{k \neq i} \sum_{j \neq i,k} a_j a_k P_{ij} P_{ik} \right)$$

$$= \left(\frac{M-N}{M-1}\right) N \left(a_i^2 P_{ii}\left(1-P_{ii}\right) + \sum_{j \neq i} a_j^2 P_{ij}\left(1-P_{ij}\right) - 2\sum_{j \neq i} a_i a_j P_{ii} P_{ij} - 2\sum_{k \neq i} \sum_{j \neq i,k; j<k} a_j a_k P_{ij} P_{ik} \right)$$

$$=\left(\frac{M-N}{M-1}\right)N\left(4P_{ii}\left(1-P_{ii}\right)+\sum_{j\neq i}P_{ij}\left(1-P_{ij}\right)-2\sum_{j\neq i}\left(2P_{ii}\right)P_{ij}-2\sum_{k\neq i}\sum_{j\neq i,k;j<k}P_{ij}P_{ik}\right)$$

$$=\left(\frac{M-N}{M-1}\right)N\left(4P_{ii}-\left(2P_{ii}\right)^2+\sum_{j\neq i}P_{ij}-\sum_{j\neq i}P_{ij}^2-2\sum_{j\neq i}\left(2P_{ii}\right)P_{ij}-2\sum_{k\neq i}\sum_{j\neq i,k;j<k}P_{ij}P_{ik}\right)$$

$$=\left(\frac{M-N}{M-1}\right)N\left(\left(2P_{ii}+\sum_{j\neq i}P_{ij}\right)-\left(\left(2P_{ii}\right)^2+\sum_{j\neq i}P_{ij}^2+2\sum_{j\neq i}\left(2P_{ii}\right)P_{ij}+2\sum_{k\neq i}\sum_{j\neq i,k;j<k}P_{ij}P_{ik}\right)+2P_{ii}\right). \quad \text{(S4)}$$

$p_i = P_{ii}+\sum_{j=1,j\neq i}^{n}\left(P_{ij}/2\right)$, and thus $2P_{ii}+\sum_{j\neq i}P_{ij}=2p_i$. In addition, using the multinomial series [2]:

$$\left(2p_i\right)^2=\left(2P_{ii}+\sum_{j\neq i}P_{ij}\right)^2=\left(2P_{ii}\right)^2+\sum_{j\neq i}P_{ij}^2+2\sum_{j\neq i}\left(2P_{ii}\right)P_{ij}+2\sum_{k\neq i}\sum_{j\neq i,k;j<k}P_{ij}P_{ik}. \quad \text{(S5)}$$

Thus, (S4) can be simplified to

$$\sigma^2\left[Y_{i,N}\right]=\left(\frac{M-N}{M-1}\right)N\left(2p_i-4p_i^2+2P_{ii}\right). \quad \text{(S6)}$$

Hence,

$$\sigma^2\left[p_{i,N}\right]=\frac{\sigma^2\left[Y_{i,N}\right]}{\left(2N\right)^2}=\frac{\left(M-N\right)\left(p_i-2p_i^2+P_{ii}\right)}{2\left(M-1\right)N}, \quad \text{(S7)}$$

which is equivalent to equation (11).

References for Supporting Information S1

1. Johnson NL, Kotz S, Balakrishnan N (1997) Discrete multivariate distributions. Hoboken, USA, Wiley-Blackwell. 328 p.

2. Weisstein, EW. “Multinomial Series.” From MathWorld--A Wolfram Web Resource. http://mathworld.wolfram.com/MultinomialSeries.html
